# The Impact of PTSD on Functioning in Patients Seeking Treatment for Chronic Pain and Validation of the Posttraumatic Diagnostic Scale

**DOI:** 10.1007/s12529-017-9641-8

**Published:** 2017-02-13

**Authors:** Sophia Åkerblom, Sean Perrin, Marcelo Rivano Fischer, Lance M. McCracken

**Affiliations:** 1 Department of Pain Rehabilitation, Skåne University Hospital, Lund, Sweden; 2 Department of Psychology, Lund University, Lund, Sweden; 3 Department of Health Sciences, Lund University, Lund, Sweden; 4 Psychology Department, Health Psychology Section, King’s College London, London, UK

**Keywords:** Chronic pain, Posttraumatic stress disorder, Pain-related functioning, The posttraumatic diagnostic scale

## Abstract

**Purpose:**

The purpose of this study was to assess the psychometric properties of a Swedish version of the Posttraumatic Diagnostic Scale (PDS); to investigate the prevalence of traumatic experiences, trauma types, and posttraumatic stress disorder (PTSD) in a sample of patients seeking treatment for chronic pain; and to examine how indices of pain-related functioning vary with a history of traumatic exposure and PTSD diagnostic status.

**Method:**

Participants were 463 consecutive patients with chronic pain referred for assessment at the Pain Rehabilitation Unit at Skåne University Hospital.

**Results:**

The translated version of the PDS demonstrated high levels of internal consistency and a factor structure similar to that reported in previous validation studies using samples identified because of trauma exposure (not chronic pain), both of which provide preliminary support for the validity of this translated version. Based on their responses to the PDS, most patients (71.8%) reported one or more traumatic events with 28.9% fulfilling criteria for a current PTSD diagnosis. The patients with PTSD also reported significantly higher levels of pain interference, kinesiophobia, anxiety, and depression and significantly lower levels of life control, compared to patients exposed to trauma and not fulfilling criteria for PTSD and patients with no history of traumatic exposure.

**Conclusion:**

Consistent with previous research, a significant proportion of patients seeking treatment for chronic pain reported a history of traumatic exposure and nearly one third of these met current criteria for PTSD according to a standardized self-report measure. The presence of PTSD was associated with multiple indictors of poorer functioning and greater treatment need and provides further evidence that routine screening of chronic pain patients for PTSD is warranted. Self-report measures like the PDS appear to be valid for use in chronic pain samples and offer a relative low-cost method for screening for PTSD.

## Introduction

Chronic pain is often defined as any pain that lasts more than 3 months [1], and chronic pain of moderate to severe intensity occurs in 19% of adults in Europe [2]. Individuals presenting for treatment of chronic pain often suffer from a range of comorbid conditions, including but not limited to anxiety and depression [3–5]. One such condition, underinvestigated in this population, is posttraumatic stress disorder (PTSD), which is defined by the presence of intrusions, avoidance, negative alterations in cognitions and mood, and hyperarousal and reactivity tied to the experience of a traumatic event [6]. The prevalence of PTSD in the general population has been estimated at approximately 6.8% lifetime and 3.5% in any 12-month period [7, 8]. People seeking treatment for chronic pain experience PTSD at much higher rates with estimates ranging from 9.5 to 45.3% [9–12]. Variability in PTSD prevalence in pain samples reflects differences in sample size (and methods of ascertainment) and the PTSD criteria used for diagnosis [10]. Likewise, high rates of chronic pain have been reported in individuals identified because of PTSD, where research mainly has been carried out with veteran samples [13–15].

Chronic pain patients with PTSD have been found to score more highly on measures of emotional distress, pain intensity, and pain disability compared to those with traumatic exposure without PTSD and those without traumatic exposure [16]. The presence of PTSD in chronic pain samples is associated with a more severe presentation including higher levels of self-reported physical health problems [13], kinesiophobia [17], pain intensity [16, 18–21], pain-related disability [16, 19, 20, 22], and emotional distress [16, 18, 20, 22] and lower self-reported life control [23]. All of these variables are known to impact the efficacy of treatments for chronic pain [24–26]. Different models have been developed to explain this relationship, and two influential models are the shared vulnerability and mutual maintenance models [27, 28]. A review of these models is beyond the scope of this article. Briefly, the high rate of comorbidity between PTSD and chronic pain may reflect shared predisposing factors to develop both conditions and/or interaction of the two conditions through various mechanisms to increase the severity and duration of both. For example, pain symptoms may trigger frequent traumatic intrusions; anxious arousal and hypervigilance associated with PTSD may heighten the person’s perception of pain and promote avoidance of pain-related situations. However, the relationship between PTSD and pain-related functioning in samples of patients seeking treatment for chronic pain remains underinvestigated [21]. Undiagnosed symptoms of PTSD in people seeking treatment for chronic pain may negatively impact their prognosis, and it is to be expected that clinicians will be required to adjust their treatment protocols to optimize treatment effects for this group. Thus, it has been argued that individuals seeking treatment for chronic pain should be routinely screened for PTSD [28]. Self-report measures can be an inexpensive first step in PTSD screening, and a large number of measures are available, however few have been adequately validated in chronic pain samples. The self-report Posttraumatic Diagnostic Scale (PDS) developed by Foa and colleagues [29] is among the most widely used measures of PTSD. The PDS is a comprehensive measure assessing the history of trauma (including the event that is/was most distressing), all of the DSM-IV symptoms of PTSD as well as their severity, the duration of symptoms, and associated impairment. The psychometric properties of the PDS have been fully reported [30–36] but not yet on a Swedish language version of the scale.

The aims of this study are threefold. The first aim is to assess the psychometric properties of a Swedish version of the PDS. First, we investigate whether the PDS possesses acceptable internal consistency and construct validity. To assess the latter, we examine the correlations between the PDS and measures assessing convergent constructs. For reasons relating to measurement burden on a clinical group, another trauma/PTSD measure was not administered. Instead, we examine the relationship between scores on the PDS and measures of anxiety, depression, and kinesiophobia (fear of movement that might exacerbate pain), which are used as part of the standard clinical assessment of adults seeking treatment for chronic pain at the unit where the present data were collected. Consistent with previous literature [29, 31, 34, 35, 37], and given that the PTSD criteria include symptoms of arousal, avoidance, fearful preoccupation, low mood, negative beliefs, concentration difficulties, and sleep disturbance, we anticipate moderately sized correlations between the PDS and measures of anxiety, depression, and kinesiophobia.

As a further examination of the validity of this Swedish translation of the PDS, we undertake confirmatory factor analyses to assess the structural validity of the PDS. Several studies have investigated the factor structure of the symptom portion (part 3) of the PDS and concluded that the observed factors do not reliably correspond to the three symptom clusters described in DSM-IV, i.e., re-experiencing, avoidance/numbing, and hyperarousal [32, 36, 38]. In fact, evidence from factor analytic investigations suggests that the three symptom clusters from the DSM-IV may not conceptualize PTSD symptoms in an optimal way [39]. Instead, the four-factor models of emotional numbing [40] and dysphoria [41] have gained empirical support along with other models. These four-factor models influenced the most recent revision of the DSM (5th edition), which now subdivides the PTSD symptoms into four clusters. Notably, the PTSD symptom criteria in DSM-5 more closely resemble the four-factor structure as suggested by the emotional numbing model [42, 43], i.e., re-experiencing (factor 1), avoidance (factor 2), emotional numbing (factor 3; similar to the negative cognitions and mood factor from the DSM-5), and hyperarousal (factor 4; similar to the alterations in arousal and reactivity factor from the DSM-5). So in a partial test of the structural validity of this Swedish translation of the PDS, we test whether the four-factor emotion numbing model, comparable to the DSM-5 symptom clusters, better fits the data than the three-factor solution corresponding to the DSM-IV PTSD criteria.

The second aim is to investigate the prevalence of traumatic experiences, trauma types, and PTSD in a sample of patients seeking treatment for chronic pain. Based on previous research [10, 16], we anticipated that ≥60% would report a history of traumatic exposure and ≥20% would report symptoms consistent with a diagnosis of PTSD. The third aim is to examine how indices of pain-related functioning vary with a history of traumatic exposure and PTSD diagnostic status, as assessed by the PDS. Differences in clinical characteristics between three groups of patients with chronic pain are explored: those fulfilling criteria for PTSD, those exposed to trauma and not fulfilling criteria for PTSD, and those not exposed to trauma. Based on previous research [13, 16–23] and informed by the mutual maintenance model [27], we anticipated that indices of pain, mental health, and general health would all show greater dysfunction in the PTSD group versus the other groups and that there would be no differences between the trauma-exposed group with no PTSD and the group with no history of traumatic exposure.

## Materials and Methods

### Participants

Participants were 463 adults with chronic pain consecutively referred for assessment at the Pain Rehabilitation Unit at Skåne University between August 2013 and February 2015. This government-funded unit based within Swedish national health services is the largest specialist center for the assessment and treatment of chronic pain in Region Skåne, the southernmost region of Sweden with 1.26 million inhabitants (approximately 13% of the total Swedish population). All patients gave written informed consent prior to their data being used in the study, and the study was approved by the Regional Ethical Review Board (2013/381).

The sample consisted of 334 women (72.1%) and 129 men (27.9%) between 18 and 67 years of age (M = 41.1 years; SD = 11.3). The majority (77.4%) were born in Sweden or another Nordic country, and approximately half (53.3%) were currently in work or further education. More than half had upper secondary school as their highest level of education (54.4%), with 14.2% having completed secondary school and 27.1% having studied at or completed university. The median time since pain onset was 5.0 years (range = 29.3 years), and the median number of pain locations was 13.0 (range = 36 locations). Usual pain intensity rated on a 0–10 scale was 7.5 (SD = 1.5). The most frequently reported primary diagnoses were fibromyalgia (28.4%), cervicocranial syndrome (9.6%), cervicobrachial syndrome (8.7%), lumbago (8.7%), and myalgia (7.0%). The sociodemographic and clinical characteristics of this sample were similar to the clinic’s referrals as a whole and to patients seeking treatment for chronic pain at other regional specialist pain units across Sweden [44].

### Translation of the PDS

Internationally recommended guidelines were followed when translating the PDS for the purposes of this study [45]. First, a translation was made by a Swedish clinical psychologist who was fluent in English and with detailed knowledge of the research field. Second, the PDS was back-translated into English by a Swedish psychologist who was also fluent in English and with experience of instrument translation and validation. Third, the translated and back-translated versions of the PDS were reviewed by an expert group working in the field of pain rehabilitation with specific knowledge of PTSD and fluent in both Swedish and English. Changes and updates were made according to suggestions of the expert group. The updated version of the PDS was then administered to a group of patients from the pain clinic to identify any needed changes to improve clarity and comprehensibility of the instructions and items, and then a final version was agreed by the expert group.

### Measures

Prior to the first face-to-face assessment at the chronic pain unit, a range of self-report measures (described below) were sent to the patients’ homes to be completed and brought to their first full clinical assessment (functioning, suitability for treatment, and diagnosis). At the same time, the patients also provided information relating to age, gender, education, country of birth, work status, pain sites, and pain duration.

#### The Posttraumatic Diagnostic Scale

The PDS is a 49-item self-report measure of current PTSD symptoms related to a single identified traumatic event. It assesses all of the diagnostic criteria (A–F) for PTSD in DSM-IV and is composed of four parts. Part 1 is a trauma checklist including serious accident, natural disaster, non-sexual assault by someone you know, non-sexual assault by a stranger, sexual assault by someone you know, sexual assault by a stranger, combat, sexual contact when young with someone 5 years older, imprisonment, torture, life-threatening disease, and “other” traumatic event (not captured by the categories). Part 2 asks the respondents to identify their most upsetting traumatic event, when it happened, if anyone was injured, if they perceived life threat, and whether the event resulted in helplessness or terror. Part 3 measures the severity of the 17 symptoms listed under categories B (intrusive recollections), C (avoidance/numbing), and D (hyperarousal) in DSM-IV. Part 4 measures interference from these 17 symptoms. A total severity score (ranging from 0 to 51) is calculated based on the 17 symptoms in part 3 (1–10 = mild; 11–20 = moderate; 21–35 = moderate to severe; ≥36 = severe). The original scale has been found to possess excellent internal consistency (*α* = .92), satisfactory test-retest coefficients (range = .77 to .85), and good convergent validity in relation to PTSD diagnosis assessed by standardized clinical interview. The scale has also demonstrated satisfactory relationships with measures of anxiety and depression [29]. The reliability and validity of a translated Swedish version were tested in this study.

#### Hospital Anxiety and Depression Scale

The HADS is a 14- item self-report measure designed to identify symptoms of anxiety and depression (separately) among patients in a medical setting. The anxiety and depression subscales each contain seven items rated on a four-point scale (0 to 3) with higher scores indicating greater severity. Consistent with the original [46], the Swedish version used in this study has excellent internal reliability for the total (*α* = .90), anxiety (*α* = .84), and depression scales (*α* = .82). It also correlates significantly with alternative measures of anxiety and depression [47].

#### The Medical Outcomes 36-Item Study Short Form Health Survey

The 36-Item Study Short Form Health Survey (SF-36) is a 36-item self-report measure of non-disease-specific health and functioning widely used in health research. It is composed of 8 subscales, but only physical functioning (10 items) and general health (5 items) were used in this study. Scores on the subscales are transformed to a 0–100 scale with higher scores indicating a greater health state. Consistent with the English language original [48], the Swedish version used in this study has been shown to have satisfactory internal reliability for the subscales (*α* = .79 to .91) and clinical validity when compared with other measures of health functioning [49].

#### Multidimensional Pain Inventory (Version 2)

The Multidimensional Pain Inventory (MPI, version 2) is a 61-item measure of pain-related functioning. Items are broken down into three sections, each composed of its own subscales. Only two subscales of the first section were used in this study: pain interference (11 items) and life control (4 items). Items are rated on a 7-point scale (0 *=* never; 6 = very often) [50]. The original MPI has satisfactory psychometric properties (*α* = .70 to .90) [51], and the Swedish version used in this study correlates significantly with other measures of pain-related functioning and is sensitive to the effects of treatment targeting chronic pain [24, 52].

#### Numerical Rating Scale

The Numerical Rating Scale (NRS) is the most widely used measure of pain intensity in both research and clinical settings. It is composed of a single item wherein the patient rates the intensity of their pain over the past week on a scale ranging from 0 (no pain) to 10 (worst possible pain)*.* The NRS is commonly used in pain clinics and research and been shown to be a valid and sensitive measure of pain intensity [53, 54].

#### Tampa Scale of Kinesiophobia

The Tampa Scale of Kinesiophobia (TSK) is a 17-item self-report measure of fear of movement or (re)injury. Items are rated on a 4-point scale (1 = strongly disagree; 4 = strongly agree) with higher scores indicating greater fear of movement/(re)injury. The TSK, including the Swedish version used in this study, has been shown to have satisfactory psychometric properties (*α* = .67 to .76) [37, 55, 56] and to correlate significantly with measures of pain-related functioning and with measures of anxiety and depression [37, 56, 57].

### Statistical Approach

#### Psychometric Analyses

The internal consistency and reliability of the PDS were examined via inter-item correlations, item-total correlations, and Cronbach’s alpha. Item-total correlations above *r* = .30 and a Cronbach’s alpha above .70 were considered acceptable [58]. Construct validity was assessed via pairwise Pearson correlations between the PDS, total score and total scores on each of the subscales, total scores on the anxiety and depression subscales of the HADS, and total score on the TSK. Correlations between the PDS, total score and total scores on each of the subscales, and relevant clinical variables (pain duration, number of pain locations, pain intensity, pain interference, life control, physical functioning, and general health) were also investigated. Correlation coefficients were evaluated as small (.1–.3), moderate (.3–.5), or strong (.5–1.0) [59].

Confirmatory factor analyses were undertaken using maximum likelihood estimation procedures with goodness of fit evaluated by the relative/normed chi-square statistic (*χ* ^2^/df; acceptable fit <5) [60], the root mean square error of approximation (RMSEA; close fit <.05, reasonable fit <.08, and mediocre fit <.10) [61], and the comparative fit index (CFI; adequate fit >.90, good fit >.95) [60, 62]. The relative/normed chi-square (*χ* ^2^/df) was used instead of the traditional chi-square value together with the associated *p* value because it reduces the likelihood of falsely rejecting the model when one uses large sample sizes as was done here [60]. Item loadings were investigated, and standardized regression weights above .32 were considered acceptable [63]. The sample size of the study was deemed satisfactory according to established guidelines [64, 65].

#### Descriptive Analyses

The prevalence of trauma, trauma type, and a current PTSD diagnosis were examined. Next, a multivariate analysis of variance (MANOVA) was conducted to test whether a linear combination of clinical characteristics (pain duration, number of pain locations, pain intensity, pain interference, life control, physical functioning, general health, anxiety, depression, and kinesiophobia) differed between three subgroups of chronic pain patients: group 1 = those fulfilling criteria for PTSD; group 2 = those exposed to trauma and not fulfilling PTSD criteria; and group 3 = those with no history of traumatic exposure. Patients were divided into these groups using all the DSM-IV criteria for PTSD (A to F) as measured by the self-report measure PDS. Univariate analyses of variance (ANOVAs) on each of the dependent variables or clinical characteristics were conducted as follow-up tests to the MANOVA. Post hoc analyses were performed to examine mean differences across the three groups on all clinical characteristics. Bonferroni-corrected *p* values were used to test for significance to reduce the likelihood of type I error [66]. All analyses were carried out using SPSS and AMOS (Version 22).

## Results

### Attrition Analyses

Of the 463 patients included in the study, a total of 24 (5%) had enough missing items on the PDS such that it was not possible to determine if they currently met the DSM-IV criteria for PTSD, and they were excluded from all further analyses. For the remaining 439 patients included in the descriptive analyses, percentage of missing data for clinical variables was very low (range = 0 to 4.8%) and Little’s MCAR test was non-significant (chi-square = 79.3, df = 87, *p* = .71), indicating that the data were missing at random. For the purposes of validating the PDS, only the 315 patients who reported a traumatic event on part 1 of the PDS were used in the analyses. For this subsample of trauma-exposed patients, the percentage of missing data was again very low (range = 0 to 4.8%), and Little’s MCAR test was non-significant (chi-square = 654.5, df = 625, *p* = .201), indicating that the data were missing at random. Consequently, it was deemed acceptable to use data from all patients and to impute missing values using the expectation-maximization (EM) method [67]. Visual inspection of histograms, normal Q-Q plots, and boxplots ensured that items were approximately normally distributed. Minor non-normality was seen among the items from the PDS but with all absolute values of skewness ≤1.4 and kurtosis ≤1.6. Maximum likelihood (ML) estimation is robust with respect to minor non-normality, skewness levels below 3 and kurtosis levels below 10 [68]. Therefore, ML estimation was used in the confirmatory factor analyses. Outliers (*n* = 21) were identified by computing standardized scores using absolute *z* values larger than 3 as a cutoff. The affected values of the outliers were winsorized and included in all analyses [69].

### Psychometric Analyses

#### Item Analyses

The response rates were above 95.6% for all 17 symptom severity items. All items correlated with each other at an acceptable level (*r* = .20 to .73), and item-total correlations were consistently ˃.30 analyzing all symptom severity items together (range = .45 to .71). Cronbach alphas for the total score (based on symptom items only) was high (*α* = .92). The Cronbach alphas for the items comprising the three-factor model (corresponding to DSM-IV criteria) were as follows: factor 1 (re-experiencing), *α* = .89; factor 2 (avoidance/numbing), *α* = .85; and factor 3 (hyperarousal), *α* = .82. Cronbach alphas for the emotional numbing four-factor model (similar to DSM-5 PTSD criteria) were as follows; factor 1 (re-experiencing), *α* = .89; factor 2 (avoidance), *α* = .72; factor 3 (emotional numbing), *α* = .82; and factor 4 (hyperarousal), *α* = .82. The mean total score on the PDS was 15.58 (SD = 11.72) for the entire sample and 25.36 (SD = 9.61) for patients who met criteria for a current diagnosis of PTSD, corresponding to the moderate to severe range of symptom severity. The mean total score for patients who reported a trauma but did not meet current PTSD criteria was 8.97 (SD = 7.71), corresponding to the mild range of symptom severity.

#### Validity

Correlations between PDS total score (symptom severity) and total scores on each of the three factor-derived subscales ranged from *r* = .60 to.86, and correlations between the PDS total score and total scores on each of the four factor-derived subscales ranged from *r* = .51 to .87 (all *p* < .001). Table 1 presents correlations between the PDS, total score and the total scores on the three factor-derived subscales, and convergent construct as well as clinical variables. In line with expectations, positive correlations in the moderate range were found between the PDS and anxiety, depression, and kinesiophobia. Small correlations were seen between the PDS and number of pain locations, pain intensity, pain interference, life control, physical functioning, and general health. Taken together, the results indicate acceptable construct validity of the PDS.

**Table 1:** Correlations between the PDS and clinical variables

	PDS total	Re-experiencing	Avoidance/numbing	Hyperarousal
Pain duration	−.05	−.04	−.05	−.01
Number of pain sites	.10	.13*	.04	.12*
Pain intensity	.20**	.20**	.17**	.17**
Pain interference	.28**	.22**	.32**	.23**
Life control	−.32**	−.29**	−.30**	−.29**
Physical functioning	−.22**	−.22**	−.19**	−.16**
General health	−.23**	−.22**	−.19**	−.20**
Anxiety	.49**	.47**	.41**	.42**
Depression	.40**	.34**	.42**	.36**
Kinesiophobia	.38**	.27**	.36**	.32**

Notes: Similar correlations were obtained using the four symptom clusters from the emotional numbing model. Pain intensity was assessed with the Numerical Rating Scale, pain interference and life control with the Multidimensional Pain Inventory, physical functioning and general health with the Medical Outcomes Study Short Form 36-Item Health Survey, anxiety and depression with the Hospital Anxiety and Depression Scale, and kinesiophobia with the Tampa Scale of Kinesiophobia

*p < .05; **p < .01

Fit indices from the confirmatory factor analyses are displayed in Table 2. The three-factor model based on the DSM-IV symptom criteria displayed an unfavorable fit (second column of Table 2), with mostly non-satisfactory values (RMSEA and CFI). The standardized factor loadings varied from .48 to .81. The four-factor emotional numbing model displayed a mixture of both satisfactory (CFI and *χ* ^2^/df) and unsatisfactory fit values (RMSEA) (third column of Table 2). The standardized factor loadings varied from .45 to .83. Modification indices from the emotional numbing model indicated a high level of covariance between item 37 (“Being overly alert (for example checking to see who is around you, being uncomfortable with your back to a door, etc.)) and item 38 (“Being jumpy or easily startled (for example when someone walks up behind you”)). To investigate if model fit could be improved, one complementary model was tested, where the error terms of these items were allowed to covary. This adjustment was considered appropriate since both items targeted hypervigilance and loaded on the same factor. Permitting this covariance improved the four-factor model fit and all values fell in the target range (last column of Table 2), and the standardized factor loadings varied from .44 to.83.

**Table 2:** Fit indices for three- and four-factor models of the PDS

Model	DSM-IV three-factor model	Emotional numbing four-factor model	Emotional numbing four-factor model with modifications
*χ* ^2^/df	4.17	3.39	2.53
RMSEA (confidence interval)	.10 (.09–.11)	.09 (.08–.10)	.07 (.06–.08)
CFI	.87	.91	.94

Notes: Goodness-of-fit indices were the relative/normed chi-square statistic (*χ* ^2^/df; acceptable fit <5), the root mean square error of approximation (RMSEA; close fit <.05, reasonable fit <.08, and mediocre fit <.10), and the comparative fit index (CFI; adequate fit >.90, good fit >.95)

### Descriptive Analyses

#### Prevalence of PTSD and Primary Traumatic Events

Out of 439 patients in the study, 315 (71.8%) reported experiencing at least one previous trauma(s). Of these trauma-exposed patients, a total of 127 fulfilled DSM-IV criteria (A–F) for PTSD based on their responses to the PDS (group 1 in Table 3). Thus, of the 439 patients referred for assessment of chronic pain, 28.9% fulfilled criteria for a PTSD diagnosis. A total of 188 trauma-exposed patients did not report sufficient symptoms on the PDS to fulfill DSM-IV criteria for a diagnosis of PTSD (group 2 in Table 3). For patients reporting a history of traumatic exposure (groups 1 and 2), no significant differences were observed for the types of traumas reported. When the patients were asked to identify their most upsetting traumatic event, the most frequently reported traumas were “other” traumatic event (27.9%) followed by serious accident (23.8%), life-threatening illness (8.3%), sexual assault by someone you know (7.6%), sexual assault by a stranger (4.4%), and non-sexual assault by a stranger (4.1%). Additionally, 14.6% of the patients were unable to identify their most upsetting traumatic event and instead reported multiple traumas. Finally, 124 of the 463 patients did not report a traumatic experience (group 3 in Table 3) and so no PTSD scores were calculated. Significant differences on sex or age were not seen among the three groups.

**Table 3:** Differences in clinical characteristics between patients fulfilling criteria for PTSD (group 1), trauma-exposed patients not fulfilling criteria for PTSD (group 2), and non-trauma exposed patients (group 3)

Variable	*F*	M (sd)			Mean differences		
		Group 1 (*n* = 127)	Group 2 (*n* = 188)	Group 3 (*n* = 124)	1 vs 2	1 vs 3	2 vs 3
Pain duration	.22	7.24 (7.32)	7.74 (7.32)	7.78 (7.50)	−.50	−.54	−.04
Number of pain sites	3.61*	15.93 (9.14)	15.24 (9.02)	13.08 (8.28)	.68	2.85*	2.16
Pain intensity	3.59*	7.67 (1.56)	7.24 (1.54)	7.56 (1.39)	.43*	.11	−.33
Pain interference	9.19**	5.00 (.88)	4.59 (.91)	4.59 (.94)	.41**	.41**	−.00
Life control	12.61**	1.89 (1.05)	2.47 (1.14)	2.47 (1.05)	−.58**	−.57**	.01
Physical functioning	4.73**	42.53 (24.03)	50.20 (21.25)	48.71 (21.88)	−7.67**	−6.17	1.50
General health	3.82*	32.62 (17.18)	37.60 (18.95)	38.42 (18.92)	−4.99	−5.80*	−.82
Anxiety	33.49**	13.46 (4.38)	9.66 (4.52)	9.40 (4.71)	3.80**	4.06**	.26
Depression	17.75**	11.81 (4.40)	9.14 (4.43)	8.92 (4.40)	2.67**	2.89**	.22
Kinesiophobia	13.32**	46.16 (9.21)	41.08 (9.02)	41.55 (8.92)	5.08**	4.61**	−.47

Notes: All alpha levels were adjusted using the Bonferroni correction. Degrees of freedom were df1 = 2 and df2 = 436. Pain duration was measured in years. Pain intensity was assessed with the Numerical Rating Scale, pain interference and life control with the Multidimensional Pain Inventory, physical functioning and general health with the Medical Outcomes Study 36-Item Short Form Health Survey, anxiety and depression with the Hospital Anxiety and Depression Scale, and kinesiophobia with the Tampa Scale of Kinesiophobia

*p < .05; **p < .01

#### Group Differences

A MANOVA was conducted to test the hypothesis that there would be significant mean differences between three groups of patients: group 1 = those fulfilling criteria for PTSD, group 2 = those exposed to trauma and not fulfilling PTSD criteria, and group 3 = those with no history of traumatic exposure, on a combination of their clinical characteristics measured prior to face-to-face assessment and treatment. A statistically significant effect was obtained (Wilk’s *ʌ* = .82; *F* (20,854) = 4.36; *p* < .001), with 9% of the variance in the canonically derived dependent variable accounted for by group membership. This significant MANOVA was followed by a series of ANOVAs for each of the dependent variables using Bonferroni-adjusted alphas (see Table 3). With the exception of pain duration, significant differences were observed between the three groups for number of pain sites, pain intensity, pain interference, life control, physical functioning, general health, anxiety, depression, and kinesiophobia (all *p* ≤ .05). Post hoc comparisons using Bonferroni-adjusted alpha indicated that group 1 had significantly higher levels of pain interference, kinesiophobia, anxiety, and depression and a significantly lower level of life control when compared with groups 2 and 3. Furthermore, group 1 had significantly higher pain intensity and lower physical functioning compared to group 2 as well as worse general health and more pain sites compared to group 3. Consistent with expectations, no significant differences on any of the variables were found between groups 2 and 3.

## Discussion

The first aim of this study was to assess the psychometric properties of a Swedish version of the Posttraumatic Diagnostic Scale (PDS). Overall, this Swedish language version of the PDS demonstrated high levels of internal consistency and reliability as well as acceptable construct validity. Likewise, and in accordance with previous studies of the factorial validity of the PDS in samples selected because of exposure to trauma and not chronic pain, the three-factor solution based on the PTSD symptom clusters in the DSM-IV did not provide an optimal fit to the data [35, 36, 39]. Instead, a better fit was obtained with a four-factor solution consistent with the emotional numbing model [40], which is similar to the PTSD symptoms clusters as now defined in DSM-5 [42, 43]. Hence, the results from this study support the bifurcation of the original avoidance/numbing criterion in DSM-IV and the resulting clustering of symptoms into four separate criteria in DSM-5. Research within this field is ongoing, and other models, such as the five-factor dysphoric arousal model separating the hyperarousal symptoms into two categories, have been proposed and gained tentative empirical support [35, 42]. It is also important to note that proposals have been made to revise the existing formulation of PTSD in the upcoming ICD-11, removing all symptoms non-specific to PTSD leaving only re-experiencing, avoidance, and perception of heightened current threat [70]. Hence, further studies are needed on the clustering of PTSD symptoms. To our knowledge, this is the first study to carry out a confirmatory factor analysis of the PDS in a chronic pain sample. Our findings overall suggest that the measure performs similarly in chronic pain patients as it does in samples selected for traumatic exposure or because they were seeking treatment for PTSD. While further validation is possible particularly employing structured diagnostic interviews for PTSD, the present findings suggest that the PDS is a reliable and sound measure that is able to yield valid inferences regarding PTSD in chronic pain samples.

The second aim of this study was to investigate the prevalence of traumatic experiences, trauma types, and posttraumatic stress disorder (PTSD) in a sample of patients seeking treatment for chronic pain. Consistent with our hypotheses and previous studies of people with chronic pain [9–12, 16], we found high rates of traumatic exposure (71.8%) and of PTSD (28.9%). These findings for the prevalence of PTSD are in line with those (23%) reported in another study based in Scandinavia and focused on people with chronic pain [10]. In agreement with earlier research, “other” traumatic events, serious accidents, life-threatening disease, sexual and non-sexual assault, and multiple traumas were frequently reported traumas in this study [10, 12].

The final aim of the study was to examine how indices of pain-related functioning vary with a history of traumatic exposure and PTSD diagnostic status as assessed by the PDS. Understanding the association between PTSD and chronic pain has been identified as an important focus of research with implications for the treatment of both conditions [28]. The results from this study add to a growing body of literature which suggests that presence of PTSD symptoms may exacerbate the effects of chronic pain. This study was not designed to test mutual maintenance, nevertheless our findings might be interpreted as providing some support for such a model [27]. We found that fear of movements that might exacerbate pain (kinesiophobia) and interference from pain were elevated in the PTSD group relative to the groups without PTSD, whether they reported a history of traumatic exposure or not. It is possible that the heightened physiological arousal and hypervigilance to harm that characterize PTSD exacerbate the fear of movements associated with pain and that the avoidance of such movements is related to the greater disability and interference found in the chronic pain group with PTSD. We note however that the overall level of pain intensity was very marginally elevated and only when compared to the trauma-exposed patients without PTSD and not the non-exposed group. These findings contrast to some extent with the notion that PTSD may heighten one’s perception of pain as suggested in the mutual maintenance model [27] and with previous studies where the presence of PTSD was associated with higher pain intensity in chronic pain groups [16, 18–21]. The number of pain sites was elevated in the PTSD group relative to the non-exposed group, and no differences were observed between the PTSD and the two comparison groups for pain duration. Previous studies have not identified a relationship between PTSD and pain duration or the number of pain sites in chronic pain samples [16, 23]. Overall, the current findings point to a much clearer relationship between the presence of PTSD and certain aspects of the pain experience, i.e., the fear of movements that might trigger pain and interference from pain, rather than the duration, distribution, or intensity of pain.

In line with our hypothesis, patients with chronic pain and PTSD in this study had much poorer clinical presentations than those without PTSD. Specifically, patients with chronic pain fulfilling criteria for PTSD reported significantly higher levels of anxiety and depression and significantly lower levels of life control compared to the other groups. Furthermore, patients with PTSD reported lower levels of physical functioning compared to the trauma-exposed patients without PTSD and worse general health than the non-trauma exposed group. Overall, this study provides support for the view that PTSD symptoms interact with those of chronic pain. The direction of causality and which specific aspects of the pain experience that interact with PTSD remains somewhat unclear and requires further investigation including longitudinal designs. We did not expect there to be, nor did we find marked differences in clinical characteristics between patients with chronic pain who had been exposed to a trauma and did not meet criteria for PTSD and those with no history of traumatic exposure.

It is important to note that scores on the measure of anxiety and depression were elevated in the PTSD group compared to both comparison groups. This is consistent with previous findings [16, 22] but raises the issues of whether the exacerbation effects of PTSD on certain aspects of chronic pain arise from the cluster of symptoms unique to PTSD (i.e., intrusive recollections) or from symptoms overlapping with those of anxiety and depression, two conditions which are common in chronic pain patients [3–5]. In other words, any comorbid problem of mood and anxiety is likely to exacerbate pain-specific impairments, and the associations observed here may not be specific to the presence of PTSD. To address this question, correlations between pain-related measures and scores on the PDS re-experiencing, avoidance/numbing, and hyperarousal subscales, displayed in Table 1, can be used. Our results suggest that the correlations with pain-related functioning are equally strong among all PTSD symptom clusters. Hence, symptom clusters unique to PTSD (i.e. re-experiencing) seem to play a role in the relationship with pain-related functioning.

Current psychological treatments for chronic pain produce small to medium effect sizes, and chronic pain researchers have begun to try to identify the particular components of psychological treatments (or mechanisms) which work for different patients and to try to understand why [26, 71]. While further research is needed, the results of the present study suggest that the very modest effect sizes obtained for psychological treatment of pain may be due partly to the presence in this population of undiagnosed and untreated conditions like PTSD, whose presence may negatively influence treatments aimed at chronic pain. Considering the high prevalence of comorbid PTSD and chronic pain in specialized care research on the issue is limited. Within the PTSD literature, a few studies have examined whether empirically supported treatments are effective in treating comorbid PTSD and chronic pain. Lesser efforts have been made to investigate whether traditional pain treatments are effective in treating comorbid PTSD and chronic pain [72]. Hence, such studies are needed to address this comorbidity and identify components of psychological treatments used to target chronic pain that may be effective in reducing the adverse impacts of PTSD and improving pain-related functioning. In addition to this, studies with chronic pain patients receiving both psychological treatment for chronic pain and PTSD are needed to investigate whether concurrent or prior treatment for PTSD improves outcomes.

Findings from the present study must be viewed within the context of certain limitations. It was not possible to investigate all relevant psychometrics in this study as the standard assessment protocol of the clinic from where this sample was drawn does not include structured clinical interviews or other self-report measures of PTSD. Hence, future studies are needed to further assess the construct validity of this Swedish translation of the PDS and complement the analyses with other reliability and validity indicators. The Swedish version of the Numerical Rating Scale for pain intensity has not been formally validated but is used throughout Sweden with chronic pain patients and shows high correlations with other pain-related measures [44]. Also, the sample was composed of chronic pain patients, the majority of whom were women with relatively high levels of education, and thus, the results may not generalize to non-clinical samples, to other groups of patients who have been exposed to a trauma but are seeking treatment for some other condition, or to more diverse samples generally. There was relatively little missing data in the current study, and the data were missing at random. Nevertheless, our analyses included patients who had missing data on one or more of the studied variables and missing values were imputed using the expectation-maximization (EM) method. The Posttraumatic Diagnostic Scale for DSM-5 did not exist when data collection for this study started. Still, with the new DSM-5 and the upcoming ICD-11 diagnostic classifications for PTSD, the assessment of the DSM-IV version of the PDS can be seen as a limitation. The groups used in the analyses were divided based on a self-report questionnaire, and the participants had not been clinically diagnosed with PTSD. Still, the PDS has demonstrated high diagnostic agreement with clinical interviews for PTSD [29]. In addition to this, we lack information as to whether the index trauma for the PTSD also was the cause of the patient’s chronic pain. Finally, it is important to point out that people who are traumatically exposed but do not fulfill the diagnostic criteria for PTSD may still have symptoms of PTSD that are highly distressing or debilitating and information may be lost by looking at PTSD by using a binary variable (i.e., fulfilling criteria or not). Our analyses suggested that the chronic pain patients who reported exposure to a trauma but who did not report sufficient symptoms to meet criteria for PTSD had PTSD symptoms only in the mild range. However, in the absence of structured diagnostic interviews, it is not possible to say with certainty that all of the patients in this group did not currently meet diagnostic criteria.

In conclusion, this study supports the reliability and validity of this Swedish language version of the PDS. Consistent with previous research, a significant proportion of patients seeking treatment for chronic pain reported a history of traumatic exposure and nearly one third of these met current criteria for PTSD according to a standardized self-report measure. Patients with chronic pain and comorbid PTSD had much poorer clinical presentations than the trauma-exposed group without PTSD and the non-trauma-exposed group. The presence of PTSD was associated with multiple indictors of poorer functioning and greater treatment need and provides further evidence that routine screening of chronic pain patients for PTSD is warranted. Self-report measures like the PDS appear to be valid for use in chronic pain samples and offer a relative low-cost method for screening for PTSD.
